# Whole-Genome Mapping Reveals Novel QTL Clusters Associated with Main Agronomic Traits of Cabbage (*Brassica oleracea* var. *capitata* L.)

**DOI:** 10.3389/fpls.2016.00989

**Published:** 2016-07-06

**Authors:** Honghao Lv, Qingbiao Wang, Xing Liu, Fengqing Han, Zhiyuan Fang, Limei Yang, Mu Zhuang, Yumei Liu, Zhansheng Li, Yangyong Zhang

**Affiliations:** ^1^Key Laboratory of Biology and Genetic Improvement of Horticultural Crops, Ministry of Agriculture, Institute of Vegetables and Flowers, Chinese Academy of Agricultural SciencesBeijing, China; ^2^Key Laboratory of Biology and Genetic Improvement of Horticultural Crops (North China), Ministry of Agriculture, Beijing Vegetable Research Center, Beijing Academy of Agriculture and Forestry SciencesBeijing, China

**Keywords:** *Brassica oleracea* var. *capitata* L., agronomic traits, linkage map, QTL clusters, marker-assisted selection

## Abstract

We describe a comprehensive quantitative trait locus (QTL) analysis for 24 main agronomic traits of cabbage. Field experiments were performed using a 196-line double haploid population in three seasons in 2011 and 2012 to evaluate important agronomic traits related to plant type, leaf, and head traits. In total, 144 QTLs with LOD threshold >3.0 were detected for the 24 agronomic traits: 25 for four plant-type-related traits, 64 for 10 leaf-related traits, and 55 for 10 head-related traits; each QTL explained 6.0–55.7% of phenotype variation. Of the QTLs, 95 had contribution rates higher than 10%, and 51 could be detected in more than one season. Major QTLs included *Ph 3.1* (max *R*^2^ = 55.7, max LOD = 28.2) for plant height, *Ll 3.2* (max *R*^2^ = 31.7, max LOD = 13.95) for leaf length, and *Htd 3.2* (max *R*^2^ = 28.5, max LOD = 9.49) for head transverse diameter; these could all be detected in more than one season. Twelve QTL clusters were detected on eight chromosomes, and the most significant four included Indel481–scaffold18376 (3.20 Mb), with five QTLs for five traits; Indel64–scaffold35418 (2.22 Mb), six QTLs for six traits; scaffold39782–Indel84 (1.78 Mb), 11 QTLs for 11 traits; and Indel353–Indel245 (9.89 Mb), seven QTLs for six traits. Besides, most traits clustered within the same region were significantly correlated with each other. The candidate genes at these regions were also discussed. Robust QTLs and their clusters obtained in this study should prove useful for marker-assisted selection (MAS) in cabbage breeding and in furthering our understanding of the genetic control of these traits.

## Introduction

Selection based on breeding objects is a key step in the crop breeding process. Traditional selection mostly relies on the phenotype, i.e., the field performance of agronomic traits; this is time-consuming and costly, cannot differentiate between heterozygous and homozygous plants, and can be easily affected by the environment. In recent years, marker-assisted selection (MAS) methodology has developed quickly and is now widely used due to the advantage of high selection efficiency and co-dominance, and unlimited by the environment or plant development stage. At present, MAS has been widely used in rice (*Oryza sativa*) (Chen et al., 2001; Datta et al., 2001; Zhou et al., 2003), wheat (*Triticum aestivum*) (Singh et al., 2004), potato (*Solanum tuberosum*; Gebhardt et al., 2006), and cabbage (*Brassica oleracea*) (Chen et al., 2013; Lv et al., 2013).

Most important agronomic traits of cabbage, such as mature period, yield, plant height, and quality, show quantitative inheritance. Before the reference genome sequence of *B. oleracea* was made public in 2014 (Liu et al., 2014), QTLs were mapped to linkage groups rather than to chromosomes, using amplified fragment length polymorphism (AFLP), restriction fragment length polymorphism (RFLP), and random-amplified polymorphic DNA (RAPD) markers, etc. These aforementioned methods have been used for identification of QTLs associated with clubroot resistance (Landry, 1992; Voorrips et al., 1997; Nagaoka et al., 2010), black rot disease resistance (Camargo and Champagne, 1995), stem-related traits (Kennard et al., 1994), flowering time (Bohuon et al., 1998; Okazaki et al., 2007; Uptmoor et al., 2008), fertility (Wang et al., 2000), plant size (Lan and Paterson, 2001), regeneration capability of tissue culture in *Agrobacterium*-mediated transformation (Sparrow et al., 2004; Oldacres et al., 2005), water absorption and photosynthetic utilization efficiency (Hall et al., 2005), regeneration capability of protoplast (Holme et al., 2004), and seed germination rate under a 5% oxygen supply (Finch-Savage et al., 2005). Presently, the *B. oleracea* reference genomes of 02-12 (heading cabbage, *B. oleracea* var. *capitata*) on BRAD (http://brassicadb.org/brad/; Cheng et al., 2011) and TO1000DH (kale-like, *B. oleracea* var. *alboglabra*) on EnsemblPlants (http://plants.ensembl.org/Brassica_oleracea/Info/Index) (Parkin et al., 2014) are available, greatly facilitating cabbage QTL research: more recently, important QTLs involved in disease resistance to *Sclerotinia sclerotiorum* (Mei et al., 2013), heading traits (Lv et al., 2014), black rot resistance (Kifuji et al., 2013; Lee et al., 2015), head splitting resistance (Pang et al., 2015; Su et al., 2015), resistance to Diamondback moth (*Plutella xylostella*) (Ramchiary et al., 2015), and clubroot resistance (Lee et al., 2016) have been reported. However, numerous agronomic traits important for cabbage breeders, such as plant-type and leaf-related traits, have seldom been investigated.

In this study, we evaluated 24 main agronomic traits in three seasons based on a 196-line DH population, and for the first time in heading cabbage, mapped significant regions of QTL clusters associated with these traits and analyzed the candidate genes. These results facilitate MAS for cabbage and pave the way for a better understanding of the genetic control of these traits.

## Materials and methods

### Plant materials and field experiments

**Figure 1:**
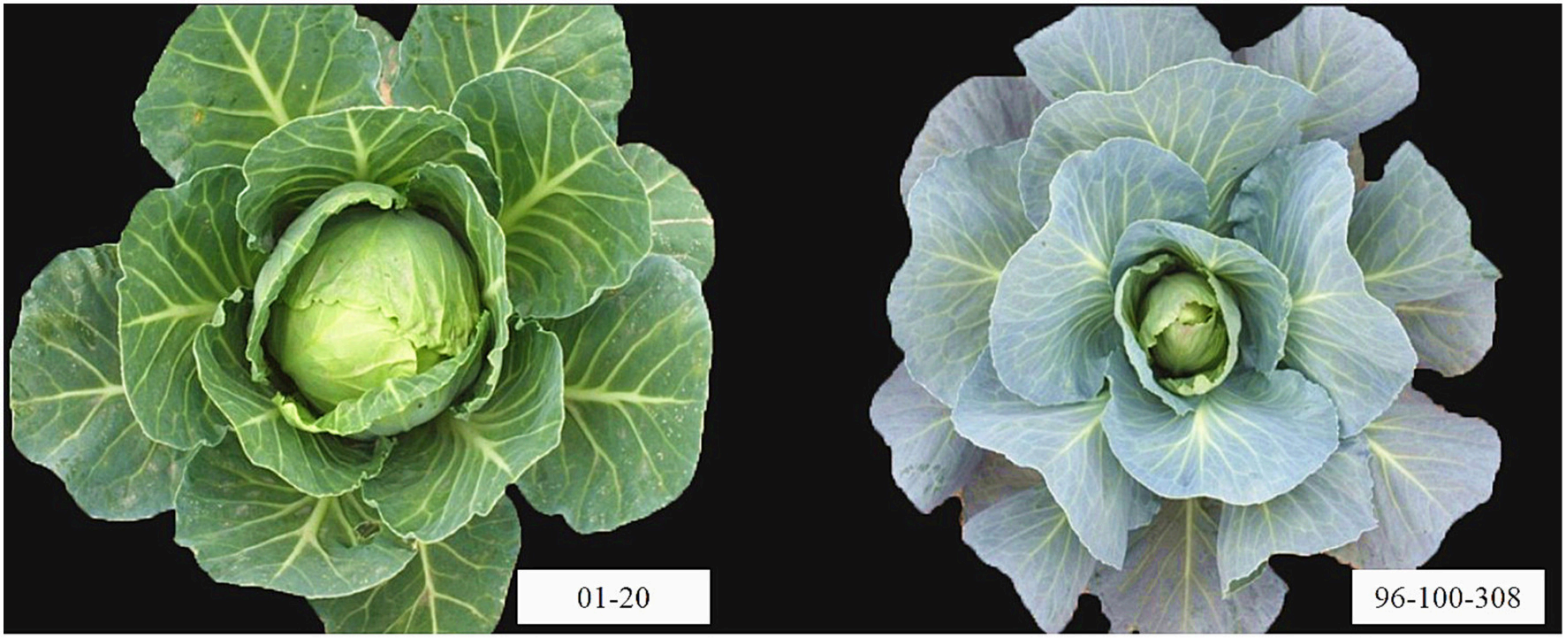
**The parental lines 01-20 and 96-100-308**. 01-20 is a spring-early-maturing inbred line with upright plant type, green leaves, little wax powder and green and round head. 96-100-308 is an autumn-late-maturing line with patulous plant type, blue leaves, thick wax power layer and slightly pointed head.

The female parental line 01-20 was bred through system selection from the conventional variety “Early Vikings” which was introduced from Canada to China in 1966 by the Institute of Vegetables and Flowers, Chinese Academy of Agricultural Sciences (IVF-CAAS). It is an early-matured spring cabbage inbred line with upright plant type, green leaves, little wax powder and green and round head; besides, 01-20 was highly susceptible to fusarium wilt, downy mildew, and black rot. The male parental line 96-100-308 was also bred through system selection from a hybrid introduced from India in 1996. This is a late-matured autumn inbred line with patulous plant type, blue leaves, thick wax power layer and slightly pointed head; besides, 96-100-308 showed strong resistance to fusarium wilt and downy mildew, and moderate resistance to black rot (Figure 1).

P_1_ (01-20) was crossed with P_2_ (96-100-308) to generate F_1_ plants, and a double haploid (DH) population consisting of 196 DH lines was obtained in 2009–2011 from the F_1_ plants through isolated microspore cultures (Takahata and Keller, 1991). These lines were also used in our previous QTL analysis of heading traits (Lv et al., 2014).

Field trials of the 196 DH lines, their parents, and F_1_ progeny were performed over three seasons at the experimental station of the IVF-CAAS, Beijing, China. The first trial, in autumn of 2011 (2011a), was conducted in an open field in Shunyi District, Beijing, China; the second in spring of 2012 (2012s) in an open field in Changping District, Beijing, China and the third in autumn of 2012 (2012a) in a greenhouse in Changping District. A randomized block design was adopted in the three seasons, with two replications. Each replication/plot consisted of 15 plants.

For spring trials, all the materials were sown on 20th January, transplanted to an open field on 20th March, and investigated from 10th May to 10th June. For autumn trials, they were sown on 20th July, transplanted to an open field on 20th August, and investigated from 10th October to 10th November.

### Data collection and statistical analysis

In total 24 main agronomic traits were measured and these traits were classified into three categories: plant-type-related traits, including plant type measured through two methods (Pt1 and Pt2, see Table 1), plant diameter (Pd), and plant height (Ph); leaf-related traits, including leaf color (Lc), leaf margin (Lm), leaf margin corrugation (Lmc), leaf surface (Ls), leaf wax powder (Lx), leaf length (Ll), leaf width (Lw), petiole length (Pl), petiole width (Pw), and leaf number (Ln); and head-related traits, including head color (Hc), head shape index (Hsi), head solidity (Hs), core width (Cw), ratio of core width to head transverse diameter (Cw/Htd), dry matter content (Dmc), and crude fiber content (Cfc).

**Table 1:** **Designation of traits and description of trait measurements**.

**Trait**	**Abb**.	**Assay time**	**Valuation criteria**
Plant type 1	Pt1	Rosette stage	Evaluation of the angle between the petiole and the horizontal plane.
Plant type 2	Pt2	Rosette stage	Visual measurement of the angle between the petiole and the horizontal plane: 1: upright; 2: half upright; 3: half patulous; 4: patulous.
Plant diameter	Pd	Harvesting stage	The maximum horizontal distance of the rosette leaves (unit: cm). Accurate to 0.1 cm.
Plant height	Ph	Harvesting stage	The distance from the plant top to the ground (unit: cm). Accurate to 0.1 cm.
Leaf color	Lc, Lca^*^, Lcb^*^ and LcL	Rosette stage	Method 1: visual measurement of the rosette leaves' color from the front side: 1: slight green; 2: green; 3: dark green; 4: slight gray green; 5: gray green; 6: dark gray green.
			Method 2: Color coordinates a^*^, b^*^, and L were measured using a CR-400 color difference meter.
Leaf wax powder	Lx	Rosette stage	General impression of the leaf wax powder: six levels were classified accordingly.
Leaf number	Ln	Harvesting stage	The number of the left leaves after head harvesting.
Leaf length	Ll	Harvesting stage	The maximum length of the largest leaf (unit: cm). Accurate to 0.1 cm.
Leaf width	Lw	Harvesting stage	The maximum width of the largest leaf (unit: cm). Accurate to 0.1 cm.
Petiole length	Pl	Harvesting stage	The maximum length of the petiole of the largest rosette leaf (unit: cm). Accurate to 0.1 cm.
Petiole width	Pw	Harvesting stage	The maximum width of the basal petiole of the largest rosette leaf (unit: cm). Accurate to 0.1 cm.
Leaf Margin	Lm	Rosette stage	Visual measurement of the margin of the rosette leaves: 1: entire margin; 2: wavy margin; 3: zigzag margin.
Leaf surface	Ls	Rosette stage	Visual measurement the surface of the rosette leaves: 1: smooth; 2: slight winkle; 3: winkle; 4: very winkle.
leaf margin corrugation	Lmc	Rosette stage	Visual measurement of the leaf margin corrugation of the rosette leaves: 1: small; 2: middle; 3: big.
Head color	Hca^*^, Hcb^*^, and HcL	Harvesting stage	Color coordinates a^*^, b^*^, and L were measured on the top of the head using a CR-400 color difference meter.
Head transverse diameter	Htd	Harvesting stage	The maximum transverse diameter of a cut-open head from the middle (unit: cm). Accurate to 0.1 cm.
Head vertical diameter	Hvd	Harvesting stage	The maximum vertical diameter of a cut-open head from the middle (unit: cm). Accurate to 0.1 cm.
Core width	Cw	Harvesting stage	The maximum transverse width of the core of a cut open head (unit: cm). Accurate to 0.1 cm.
Head shape index	Hsi	Harvesting stage	Hsi = Htd/Hvd.
Head solidity	Hs	Harvesting stage	Hs = Hw/(π/6 ^*^ Hvd ^*^ Htd^2^).
Dry matter content	Dmc	Harvesting stage	The head was cut open and sliced to 1–2 cm after removing the core and 500 g was randomly sampled and dried to constant weight (M1) at 105°C. Dmc = M1/500 ^*^ 100% (AOAC standards, 1995).
Crude fiber content	Cfc	Harvesting stage	The crude fiber content was assayed by acid digestion and alkali digestion method (AOAC standards, 1995).

Most of the traits were evaluated according to the standards described in “Descriptors and data standards for cabbage” (Li and Fang, 2007) at the rosette stage or head harvesting stage (Table 1). Besides, Dmc and Cfc were determined following drying method and acid digestion and alkali digestion method, respectively, in accordance with the AOAC standards (1995) (Table 1). For color-related traits, a CR-400 color difference meter (Konica Minolta, Shanghai, China) was used to assay leaf and head color coordinates a^*^ (redness and greenness), b^*^ (yellowness and blueness), and L (lightness) (CIE1976_Lab standards) with standard D65 light source, 0 degree/diffuse illumination and viewing angle of 2 degree to CIE 1931 under dark background. In addition, heading trait data including head maturity period (Hm), head weight (Hw), core length (Cl), head vertical diameter (Hvd), and Cl/Hvd used in our previous study (Lv et al., 2014) were used for a joint analysis, including correlation tests and QTL cluster analysis.

Three individual plants from each plot were randomly selected for data collection at the rosette or harvesting stage. Average values for each trait of each DH line were calculated from three plants in each plot. Adjusted means for the traits were obtained and used for further analysis. Microsoft Excel 2007 (Microsoft, Seattle, WA, USA) and SPSS 12.0 (SPSS, Chicago, IL, USA) software were used for statistical analyses including correlation test, analysis of variance (ANOVA), and multiple comparison. Pearson's simple correlation coefficients (*r*) were calculated between the traits, using adjusted means.

### QTL analysis for cabbage main agronomic traits

A linkage map constructed with the same DH population in our previous study (Lv et al., 2014) was used for QTL analysis. MapQTL 4.0 (Van Ooijen et al., 2002) was implemented for the QTL analysis, using interval mapping (IM) and the multiple-QTL model (MQM). Initially, 1000-permutations were performed to estimate the significance threshold of the test statistics for a QTL, based upon a 5% experiment-wise error rate. Then, interval mapping (IM) was performed every 1 cM along chromosomes to scan for QTLs with a LOD threshold of 3.0. Markers closely linked to positions with the highest LOD score were taken as cofactors for MQM analysis. Loci with the highest LOD scores were assigned as QTLs. Two-LOD-supported intervals were established as 95% confidence intervals (Van Ooijen, 1992).

QTLs were named using the following methodology: abbreviation of trait name, followed by chromosome code and QTL code. For example, *Lc 1.2* represents the second QTL on chromosome C01 for leaf color.

Meta-QTL analysis was performed with the software Biomercator v2.1 (Arcade et al., 2004), using the data obtained from MapQTL4.0. Meta-analysis was carried out separately for all chromosomes. The number of meta-QTLs present was determined as the model which minimized the Akaike criterion (AIC).

## Results

### Statistical analysis of agronomic traits with DH population

Twenty-four agronomic traits of the parental lines, F_1_, and DH population were investigated over three different environments (three seasons, two locations; e.g., 2011a, 2012s, and 2012a). The histograms showing segregation patterns were obtained for each trait using Microsoft Excel 2007 software (Supplementary Figure 1). Statistical analyses, including mean value, range, standard deviation (SD), skewness and kurtosis, and significance analysis based on least significant difference tests were performed for the trait data in all three seasons (Table 2).

Table 2**Statistical analysis of the cabbage parental lines and their F1, DH progenies**.

**Table:** 

**Traits**	**2011a**^a^								**Traits**	**2012s**							
	**P_1_**	**P_2_**	**F_1_**	**DH-11A**	**Min**.	**Max**.	**Skewness**	**Kurtosis**		**P_1_**	**P_2_**	**F_1_**	**DH-12S**	**Min**.	**Max**.	**Skewness**	**Kurtosis**
Pt1	65.01 ± 1.31a^b^	55.55 ± 0.26d	58.28 ± 0.67c	59.09 ± 0.09c	45.94	77.13	0.31	−0.45	Pt1	57.65 ± 0.5c	44.83 ± 0.85h	51.78 ± 0.42e	48.97 ± 0.18g	30.53	62.51	0.15	−0.56
Pt2	1 ± 0d	3 ± 0a	2 ± 0c	2.45 ± 0.04b	1.00	4.00	0.05	−0.63	Pt2	1 ± 0d	3 ± 0a	2 ± 0c	2.37 ± 0.04b	1	4	−0.03	−0.66
Pd	42.85 ± 1.34ef	53.21 ± 1.42b	58.13 ± 1.18a	44.57 ± 0.34de	24.92	71.50	0.34	0.87	Pd	36.72 ± 1.25g	47.92 ± 1.54cd	49.39 ± 0.78c	40.62 ± 0.06f	24.26	57.58	0.01	−0.07
Ph	26.08 ± 2.84cd	27.58 ± 1.3bc	27.2 ± 0.3bc	25.07 ± 0.23cd	15.00	50.00	0.57	−0.09	Ph	19.39 ± 1.03e	27.75 ± 1.01bc	24.56 ± 0.97cd	23.72 ± 0.05d	11.5	41	0.35	−0.47
Lc	2 ± 0e	6 ± 0a	4 ± 0c	3.48 ± 0.03d	1.00	6.00	−0.21	−0.56	Lc	2 ± 0e	5 ± 0b	4 ± 0c	4.09 ± 0.1c	1	6	−0.86	0.71
Lca^*^	−7.96 ± 0.13d	−4.38 ± 0.21a	−5.04 ± 0.01b	−56.18 ± 0.01c	−3.74	−9.73	0.60	0.30	Lca^*^	−11.79 ± 0.09h	−9.03 ± 0.26f	−11.02 ± 0.06g	−10.87 ± 0.17g	−8.29	−13.77	−0.07	−0.12
Lcb^*^	10.19 ± 0.27c	5.17 ± 0.39e	5.74 ± 0.01e	7.4 ± 0d	3.24	13.07	0.54	0.02	Lcb^*^	16.67 ± 0.21a	11.13 ± 0.58c	15.01 ± 0.08b	14.61 ± 0.04b	9.9	21.59	0.35	0.16
LcL	32.67 ± 0.38h	36.01 ± 0.07g	32.62 ± 0.15h	33.11 ± 0h	27.85	37.32	−0.37	−0.10	LcL	45.15 ± 0.04bc	48.79 ± 0.05a	44.61 ± 0.19c	46.05 ± 0.14b	40.36	54.05	0.69	0.74
Lx	2 ± 0f	5 ± 0b	4 ± 0c	3.31 ± 0.01e	1.00	6.00	−0.15	−0.63	Lx	2 ± 0f	6 ± 0a	4 ± 0c	3.94 ± 0.01c	1	6	0.42	−0.55
Ln	11.67 ± 0.58g	16.67 ± 0.01e	13.83 ± 0.29f	14.88 ± 0.05f	10.25	24.17	0.68	0.32	Ln	18.33 ± 0.84d	25 ± 0.29a	20 ± 1.2c	22.31 ± 0.12b	14.5	52.67	0.88	0.96
Ll	28.21 ± 0.84b	28.28 ± 1.13b	33.08 ± 1.49a	25.85 ± 0.28bc	15.37	41.65	0.81	0.96	Ll	21.03 ± 0.59d	24.97 ± 1.1c	26.39 ± 0.97b	21.58 ± 0.15d	12.18	32.73	0.30	0.35
Lw	23.87 ± 0.65de	26 ± 1.15cd	32.02 ± 1.16a	23.92 ± 0.23de	12.88	36.37	0.45	0.87	Lw	16.20 ± 0.43f	23.27 ± 0.99e	22.42 ± 0.31e	17.92 ± 0.12f	9.63	26.67	0.35	0.27
Pl	−	−	−	−	−	−	−	−	Pl	0e	4.8 ± 0.94bc	5.67 ± 0.62b	2.34 ± 0.03d	0	10	0.63	−0.77
Pw	−	−	−	−	−	−	−	−	Pw	2.86 ± 0.01c	3.37 ± 0.07ab	3.37 ± 0.17ab	2.85 ± 0.05c	1.4	3.73	−0.17	0.86
Lm	1 ± 0e	1 ± 0e	2 ± 0e	1.58 ± 0.01b	1.00	3.00	−0.02	−0.87	Lm	1 ± 0e	1 ± 0e	2 ± 0e	1.31 ± 0a	1	3	0.98	−0.59
Ls	1 ± 0d	1 ± 0 d	2 ± 0a	1.76 ± 0.02c	0.00	3.00	0.03	−0.28	Ls	1 ± 0d	1 ± 0d	2 ± 0a	1.96 ± 0.01b	1	3	0.46	−0.63
Lmc	0 ± 0e	0 ± 0e	0 ± 0e	0.38 ± 0b	0.00	3.00	1.89	1.95	Lmc	0 ± 0e	0 ± 0e	0 ± 0e	1.1 ± 0.01a	0	3	0.44	−1.53
Hca^*^	−17.73 ± 1.85de	−16.01 ± 0.35cd	−12.77 ± 0.74a	−15.84 ± 0.32cd	−12.77	−19.22	0.34	−0.54	Hca^*^	−17 ± 0.35de	−18.56 ± 0.14e	−17.82 ± 0.12de	−17.93 ± 0.04de	−14.81	−20.28	0.44	−0.55
Hcb^*^	27.55 ± 4.26bcde	32.73 ± 0.01ab	24.76 ± 3.84cde	28.56 ± 1.44bcd	21.13	35.30	−0.20	−0.86	Hcb^*^	30.01 ± 0.58abc	34.21 ± 0.46a	32.42 ± 0.3ab	31.15 ± 0.17ab	23.84	35.52	−0.66	−0.05
HcL	55.92 ± 1.61edf	66.2 ± 0.48a	56.51 ± 3.12cde	59.58.0.9bc1	49.36	68.22	−0.32	−0.99	HcL	57.1 ± 0.59cd	64.54 ± 0.32a	60.39 ± 0.31b	59.45 ± 0.06bc	53.34	67.52	0.46	−0.09
Htd	16.25 ± 0.55b	14.55 ± 0.26c	19.21 ± 0.5a	14.58 ± 0.13c	8.32	23.00	0.68	0.89	Htd	11.86 ± 0.12fgh	12.1 ± 0.33fg	13.12 ± 0.23de	11.65 ± 0.06gh	7.87	15.6	0.25	−0.13
Hs	0.49 ± 0.01h	0.62 ± 0.02e	0.63 ± 0.01de	0.52 ± 0.01gh	0.23	0.79	−0.15	−0.45	Hs	0.67 ± 0.01cd	0.75 ± 0.01b	0.8 ± 0.01a	0.71 ± 0.02bc	0.5	0.92	0.10	0.15
Cw	2.27 ± 0.12g	3.48 ± 0.01bc	3.23 ± 0.03d	2.69 ± 0f	1.53	3.52	−0.30	0.99	Cw	2.69 ± 0.06f	3.95 ± 0.13a	3.6 ± 0.04b	2.98 ± 0.02e	2	4.1	0.11	0.20
Cw/Htd	0.14 ± 0.01i	0.24 ± 0.01e	0.17 ± 0.01h	0.19 ± 0g	0.10	0.27	0.03	0.78	Cw/Htd	0.23 ± 0.01ef	0.33 ± 0.01a	0.28 ± 0.01c	0.26 ± 0d	0.18	0.37	0.25	−0.10
Hsi	1 ± 0.02c	1 ± 0.01c	0.87 ± 0.01d	1.08 ± 0.01b	0.67	1.61	0.67	0.58	Hsi	1.1 ± 0.02b	1.13 ± 0.02a	1.09 ± 0.01ab	1.12 ± 0.01ab	0.9	1.37	0.30	−0.21
Hs	0.49 ± 0.01h	0.62 ± 0.02e	0.63 ± 0.01de	0.52 ± 0.01gh	0.23	0.79	−0.15	−0.45	Hs	0.67 ± 0.01cd	0.75 ± 0.01b	0.8 ± 0.01a	0.71 ± 0.02bc	0.5	0.92	0.10	0.15
Dmc	−	−	−	−	−	−	−	−	Dmc	6.29	7.93	6.61	7.23	5.24	9.36		
Cfc	−	−	−	−	−	−	−	−	Cfc	0.46	0.74	0.52	0.59	0.42	0.85

**Table:** 

**Traits**	**2012a**							
	**P_1_**	**P_2_**	**F_1_**	**DH-12A**	**Min**.	**Max**.	**Skewness**	**Kurtosis**
Pt1	61.21 ± 0.85b	49.56 ± 0.63fg	51.38 ± 0.75ef	49.09 ± 0.09g	33.61	69.05	0.02	−0.49
Pt2	1 ± 0d	3 ± 0a	2 ± 0c	2.39 ± 0.08b	1	4	−0.04	−0.74
Pd	40.33 ± 0.88f	55 ± 0.99b	56 ± 1.01ab	46.87 ± 0.55d	28.33	67.25	−0.05	0.21
Ph	27 ± 1.15bcd	33 ± 0.99a	33 ± 1.15a	29.89 ± 0.29ab	17.25	48.5	0.77	0.81
Lc	2 ± 0e	6 ± 0a	4 ± 0c	4.08 ± 0.02c	1	6	−0.46	0.33
Lca*	−10.47 ± 0.2g	−5.59 ± 0.35bc	−8.19 ± 0.5de	−8.77 ± 0.07ef	−5.35	−13.07	−0.26	−0.04
Lcb*	15.76 ± 0.41ab	5.95 ± 0.67e	10.88 ± 1.1c	11.4 ± 0.01c	2.54	19.83	0.07	−0.03
LcL	38.37 ± 0.43f	41.49 ± 1.2d	39.8 ± 0.55e	40.09 ± 0.57e	35.93	46.44	0.38	0.50
Lx	2 ± 0f	6 ± 0a	4 ± 0c	3.7 ± 0.01d	1	6	0.22	−0.02
Ln	15.33 ± 0.33ef	20.67 ± 0.88c	15.33 ± 0.33ef	19.19 ± 0.11cd	11.67	33	0.76	0.42
Ll	28 ± 0.58b	31.07 ± 1.03a	31.33 ± 1.09a	26.81 ± 0.24bc	16.3	43.75	0.53	0.74
Lw	23.83 ± 1.59de	26.67 ± 0.33c	29.33 ± 1.42b	24.04 ± 0.08de	15.2	37	0.03	0.50
Pl	0e	9.73 ± 0.62a	4.03 ± 0.03c	2.53 ± 0.07d	0	13.75	0.99	−0.07
Pw	3.17 ± 0.03abc	3.47 ± 0.26a	3.33 ± 0.17ab	3.03 ± 0bc	2.13	4.1	0.19	−0.34
Lm	1 ± 0e	1 ± 0e	2 ± 0e	1.45 ± 0.01c	0	3	0.97	−0.04
Ls	1 ± 0d	1 ± 0d	1 ± 0d	1.15 ± 0d	0	3	0.51	0.79
Lmc	0 ± 0e	0 ± 0e	0 ± 0e	0.16 ± 0.01c	0	3	1.92	2.89
Hca*	−13.13 ± 0.95ab	−15.95 ± 0.53cd	−15.1 ± 0.35bc	−14.48 ± 0.02abc	−18.03	−11.25	0.15	0.10
Hcb*	22.49 ± 1.73e	30.18 ± 0.67ab	27.86 ± 0.25bcd	24.62 ± 0.33de	17.38	30.02	−0.34	−0.41
HcL	49.49 ± 0.75g	58.25 ± 0.37bcd	53.4 ± 0.22ef	52.88 ± 0.1f	47.4	59.94	0.22	−0.07
Htd	13.57 ± 0.35d	11.03 ± 0.12h	15.03 ± 0.29c	12.55 ± 0.17ef	7.55	20.25	0.57	0.96
Hs	0.57 ± 0.02fg	0.71 ± 0.02bc	0.7 ± 0.03c	0.6 ± 0.02ef	0.34	0.86	−0.16	−0.35
Cw	2.2 ± 0g	3.27 ± 0.12d	3.3 ± 0.06cd	2.86 ± 0ef	1.85	3.9	0.52	0.45
Cw/Htd	0.16 ± 0.01h	0.3 ± 0.01b	0.22 ± 0.01f	0.23 ± 0ef	0.14	0.34	0.15	−0.05
Hsi	0.99 ± 0.02c	1.11 ± 0.01ab	0.99 ± 0.01c	1.09 ± 0ab	0.76	1.46	0.63	0.30
Hs	0.57 ± 0.02fg	0.71 ± 0.02bc	0.7 ± 0.03c	0.6 ± 0.02ef	0.34	0.86	−0.16	−0.35
Dmc	−	−	−	−	−	−	−	−

*For abbreviations of the traits, see Table 1*.

a *Three seasons in which the experiment was carried out, i.e., autumn 2011, spring 2012 and autumn 2012 respectively*.

b *Values of parents, F1 and DH population were presented as: Mean ± SD. Values followed by the same letters are not significantly different at P < 0.05 level, based on LSD Test. “-” means no survey data*.

**Figure 2:**
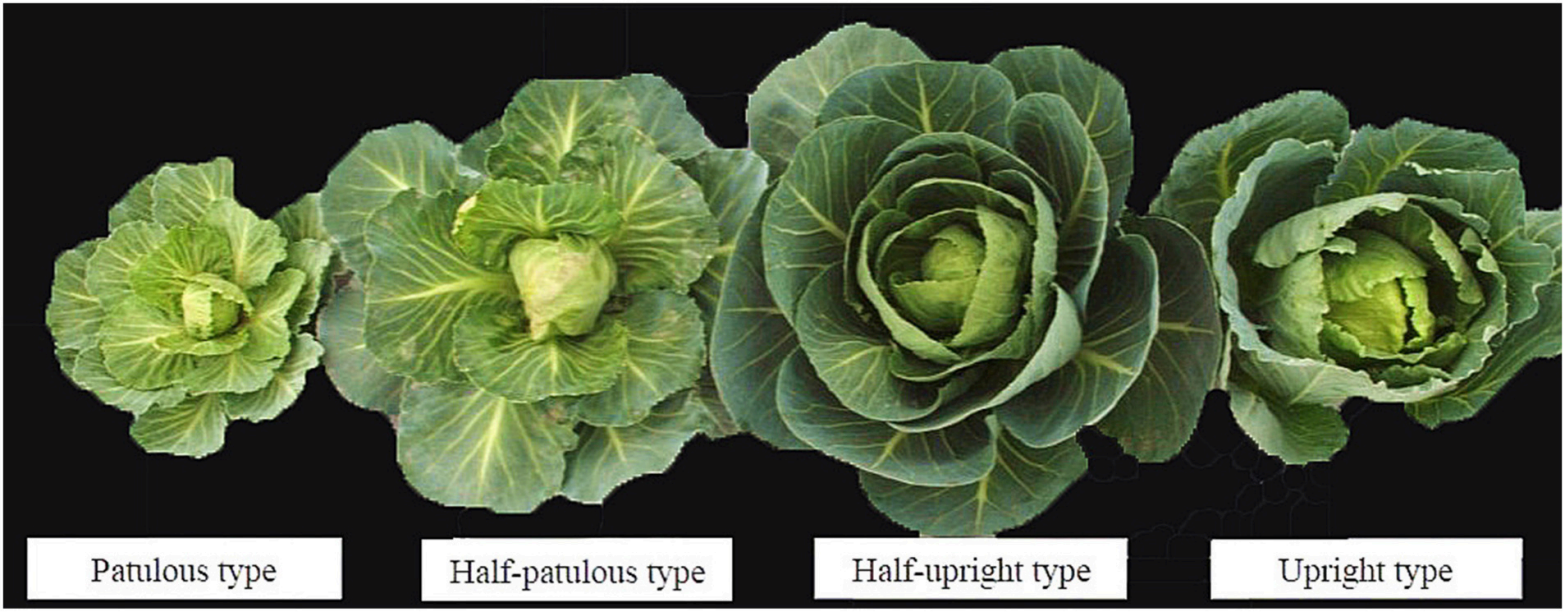
**Segregation of plant-type in the DH population**.

Some trait values for the DH population showed inter-parent variations or were similar to one parental line, while others exhibited bi-directional transgressive variations, suggesting alleles with additive effects or complementation effect for these traits were distributed among the parents. In Figure 2, the segregation of plant type in the DH population showed that some lines were more upright or patulous than the parental lines. From the skewness data it was determined that in more cases the extent of transgressive variation was toward higher rather than lower values. Skewness and kurtosis values were < 1.0 in the three data sets, with the exception of Lm and Lmc, indicating the segregation pattern of most traits generally fitted a normal or near normal distribution model suitable for QTL identification. Due to irregular segregation patterns from the histograms (Supplementary Figure 1) and higher skewness or kurtosis, Ls, Lm, and Lmc were not considered for further QTL analysis. The irregular distribution might be caused by inaccurate phenotype measurement.

The parents exhibited differences in some traits, while trait values for F_1_ plants showed inter-parent variations or were similar to one parental line. For the comparison between the two parents, no significant differences were observed between parental lines for Lm, Lmc, and Ls in all three seasons; no significant differences between parental lines were observed for Lca^*^ and Lcb^*^ in two of the three seasons; no significant differences between parental lines were observed for Ph, Ll, Lw, Pw, Hsi, and Htd in only one of the three seasons. Significant differences were observed for all other traits between the parental lines in all three seasons. For the comparison between the DH means and the parental lines, the traits for Pt1, Lca^*^, Ln, Ll, Lw, Pl, HcL, and Cw had no significant differences with P_1_ or P_2_ in one of the three seasons; the traits for plant diameter, LcL, Pw, Hca^*^, Hsi, and Hs had no significant differences with P_1_ or P_2_ in two of the three seasons; and traits for Ph, Lm, and Hcb^*^ had no significant differences with P_1_ or P_2_ in all three seasons. Most other traits showed inter-parent variations and significant differences with parental lines. For the comparison between the DH means over three seasons, no significant differences were observed for Pt and Hsi in three seasons; no significant differences were observed for Lc, Pl, Pw, and Htd in two of three seasons, and significant differences were observed for all other means of traits in three seasons.

An ANOVA test was performed to estimate the effects of season, genotype, genotype × season and block for trait data of the three seasons (Table 3). A significant (at the *P* < 0.05 level) or greatly significant (at the *P* < 0.01 level) variation among the genotypes was observed for all traits; the variation among the seasons was also significant or greatly significant for most traits except for Pt2, Lx, Pl, Lm, Ls, Lmc, Cw, his, and Hs. For all traits, no significant effect was observed for blocks, and for genotype × season, indicating that these effects were limited.

**Table 3:** **Analysis of variance for measured traits across three seasons**.

**Trait**	**Mean square**				
	**Season**	**Genotype**	**Genotype × season**	**Block (season)**	**Error**
Pt1	143.12^**a^	357.77^**^	17.41	1.22	0.05
Pt2	0.20	0.91^**^	0.28	0.01	0.02
Pd	3166.50^**^	325.99^**^	52.49	0.05	3.78
Ph	3521.99^**^	233.08^**^	30.61	2.23	4.87
Lc	10.89^**^	1.34^*^	0.33	0.001	0.01
Lca^*^	593.80^**^	10.26^*^	2.60	0.09	0.61
Lcb^*^	2635.14^**^	44.05^*^	9.31	0.37	2.00
LcL	3460.56^**^	30.38^*^	7.14	0.89	1.51
Lx	3.87	10.49^**^	0.33	0.01	0.01
Ln	2063.16^**^	81.32^**^	18.15	2.87	2.74
Ll	2116.25^**^	127.80^**^	12.77	1.58	2.46
Lw	4434.94^**^	93.62^**^	14.10	1.69	2.47
Pl	9.36	87.02^**^	9.25	1.45	1.03
Pw	1.16^**^	2.52^**^	0.17	0.02	0.05
Lm	2.66	3.88^**^	0.23	0.04	0.03
Ls	0.47	22.22^**^	0.24	0.01	0.05
Lmc	1.03	29.63^**^	0.62	0.001	0.02
Hca^*^	594.51^**^	128.05^**^	5.22	0.64	0.86
Hcb^*^	1514.36^**^	142.58^**^	20.23	1.61	3.01
HcL	1639.80^**^	252.76^**^	22.38	3.15	3.95
Htd	181.63^**^	28.23^**^	6.94	0.08	2.38
Cw	1.14	7.92^**^	0.16	0	0.03
Cw/Htd	0.47^**^	0.53^**^	0	0	0
His	0.05	0.13^**^	0.006	0	0
Hs	0.15	1.19^**^	0.004	0	0

*For abbreviation, see Table 1*.

a **Significant at P < 0.05 level,

** *Significant at P < 0.01 level*.

### Correlation analysis

Correlation analysis was performed using adjusted means of the trait data over three seasons (Table 4).

**Table 4:** **Correlation coefficients among all traits measured in the DH population**.

	**Pt1**	**Pt2**	**Pd**	**Ph**	**Lc**	Lca^*^	Lcb^*^	**LcL**	**Lx**	**Ln**	**Ll**	**Lw**	**Pl**	**Pw**	**Lm**	**Ls**	**Lmc**	Hca^*^	Hcb^*^	**HcL**	**Hvd**	**Cl**	**Cl/Hvd**	**Htd**	**Cw**	**Cw/Htd**	**Hw**	**Hsi**	**Hs**	**Hm**	**Dmc**
Pt2	−0.66^a^																														
Pd	−0.09	0.05																													
Ph	0.17	−0.23	0.74																												
Lc	−0.18	0.25	−0.06	−0.12																											
Lca^*^	−0.35	0.36	0.16	0.08	0.71																										
Lcb^*^	0.18	−0.27	−0.19	−0.18	−0.74	−0.92																									
LcL	−0.15	0.14	−0.40	−0.43	0.22	−0.14	0.07																								
Lx	−0.09	0.12	0.08	0.04	0.76	0.57	−0.67	0.27																							
Ln	−0.29	0.19	−0.09	0.13	−0.18	−0.09	0.14	0.06	−0.16																						
Ll	0.21	−0.18	0.88	0.80	−0.11	0.02	−0.12	−0.45	0.03	−0.19																					
Lw	0	−0.07	0.87	0.74	0.02	0.20	−0.27	−0.35	0.19	−0.18	0.83																				
Pl	0.19	−0.15	0.30	0.16	0.05	0.10	−0.15	−0.14	0.07	−0.36	0.38	0.27																			
Pw	−0.15	0.10	0.56	0.26	0.04	0.07	−0.06	−0.15	0.05	−0.08	0.49	0.54	0.12																		
Lm	−0.04	0.02	−0.30	−0.39	0	−0.18	0.17	0.46	−0.02	−0.08	−0.32	−0.32	0.03	−0.23																	
Ls	0.28	−0.24	0.17	0.38	−0.21	−0.14	0.08	−0.25	−0.16	0.02	0.31	0.25	0.07	0.01	−0.18																
Lmc	−0.07	0.14	−0.26	−0.44	0	−0.09	0.14	0.08	−0.21	−0.16	−0.34	−0.24	−0.06	−0.01	0.20	−0.13															
Hca^*^	0.14	−0.18	0.13	0.35	−0.02	0.01	−0.03	−0.14	0.12	0.01	0.25	0.18	0.17	−0.06	−0.16	0.12	−0.33														
Hcb^*^	−0.34	0.42	−0.24	−0.50	0.06	0.02	0.03	0.40	−0.09	0.13	−0.45	−0.30	−0.20	0.05	0.26	−0.19	0.28	−0.79													
HcL	−0.34	0.43	−0.15	−0.31	0.04	0.01	0.06	0.36	−0.05	0.22	−0.31	−0.22	−0.21	0.15	0.18	−0.20	0.10	−0.51	0.75												
Hvd	0.20	−0.20	0.63	0.69	−0.05	0.07	−0.15	−0.44	0.04	−0.08	0.74	0.55	0.13	0.37	−0.39	0.20	−0.20	0.20	−0.47	−0.42											
Cl	0.28	−0.33	0.34	0.68	−0.25	−0.20	0.12	−0.32	−0.09	0.24	0.54	0.35	−0.06	0.11	−0.38	0.33	−0.39	0.40	−0.53	−0.35	0.63										
Cl/Hvd	0.27	−0.34	0.17	0.55	−0.29	−0.27	0.21	−0.23	−0.13	0.34	0.36	0.21	−0.11	−0.01	−0.31	0.33	−0.39	0.39	−0.45	−0.27	0.34	0.94									
Htd	0.30	−0.39	0.62	0.74	−0.19	−0.15	0.04	−0.30	0.02	0.04	0.73	0.61	0.11	0.28	−0.32	0.29	−0.35	0.31	−0.50	−0.44	0.80	0.79	0.62								
Cw	−0.15	0.12	0.60	0.34	0.23	0.29	−0.36	0.02	0.33	−0.06	0.42	0.59	0.10	0.45	−0.12	−0.16	−0.10	−0.15	0.08	0.07	0.37	−0.06	−0.20	0.32							
Cw/Htd	−0.37	0.45	−0.07	−0.37	0.36	0.38	−0.34	0.30	0.26	−0.07	−0.31	−0.07	−0.02	0.09	0.20	−0.40	0.22	−0.40	0.50	0.45	−0.39	−0.71	−0.69	−0.61	0.55						
Hw	0.24	−0.3	0.63	0.73	−0.09	−0.02	−0.09	−0.31	0.08	−0.02	0.74	0.60	0.14	0.31	−0.34	0.23	−0.35	0.32	−0.52	−0.44	0.89	0.73	0.50	0.95	0.40	−0.49					
Hsi	−0.20	0.35	−0.11	−0.21	0.25	0.35	−0.29	−0.15	0.04	−0.15	−0.15	−0.21	0	0.06	−0.03	−0.19	0.27	−0.23	0.16	0.12	0.14	−0.37	−0.52	−0.47	0.01	0.43	−0.26				
Hs	0.01	−0.08	−0.07	−0.09	−0.01	−0.08	0.04	0.3	0.13	0.11	−0.15	0.01	0.02	−0.10	0.18	−0.03	−0.20	0.03	0.10	0.08	−0.43	−0.09	0.09	0	0.18	0.13	−0.03	−0.65			
Hm	−0.01	−0.13	0.60	0.77	0.06	0.18	−0.27	−0.31	0.21	0.29	0.62	0.58	0.04	0.31	−0.31	0.12	−0.41	0.36	−0.43	−0.22	0.60	0.63	0.51	0.66	0.37	−0.25	0.66	−0.18	−0.04		
Dmc	−0.26	0.22	0.26	0.26	0.28	0.44	−0.41	−0.04	0.37	0.24	0.09	0.25	−0.01	0.15	−0.11	−0.04	−0.18	0.10	−0.04	0.14	0.08	0.04	0.03	0.02	0.32	0.25	0.08	0.09	0.10	0.38	
Cfc	−0.37	0.31	0.16	0.18	0.30	0.42	−0.38	0.08	0.36	0.24	0.01	0.16	−0.07	0.16	−0.12	−0.15	−0.19	0.21	−0.05	0.22	0.06	0.04	0.03	−0.04	0.29	0.28	0.04	0.15	0.02	0.37	0.84

*For abbreviations, see Table 1*.

a *Absolution value of correlation coefficient >0.18 was considered as significant at P < 0.05 level, and >0.23 as extremely significant at P < 0.01 level*.

Little relationship was found for plant-type-related traits, except for high correlation between Pt1 and Pt2, and between Pd and Ph. Pd and Ph were considered to be significant traits, because they had significant correlations not only with most of the leaf traits, but also with almost all the head traits. Pt1 and Pt2 seemed to have low correlation with other traits.

For leaf-related traits, there was a very high correlation (>0.5; absolute value) between any two of Lc, Lca^*^, Lcb^*^, and Lx, which was in accord with the fact that a greater amount of wax powder signifies a darker leaf color. The most important leaf-related traits were Ll and Lw, who had some correlations with plant-type and leaf traits but had significant relationships with most of the head traits, indicating that they might be key selection factors in breeding.

For head-related traits, there was a high correlation (>0.5; absolute value) between any two of HcL, Hca^*^, and Hcb^*^. Hcb^*^, Hvd, and Htd were deemed as significant traits because they had high correlation with important head traits such as Hw, Hm, and Cl/Hvd. Another fact was, according to our breeding experience, although Cl had high positive correlations with Cl/Hvd (0.94), Htd (0.79), Cw/Htd (0.71), Hw (0.73), and Hm (0.63), we would rather select short core cabbage lines or cultivars considering their good commercial appearance and late-bolting character.

The highest correlations, with absolute values over 0.8, were seen between Pd and Ll (0.88), Pd and Lw (0.87), Ph and Ll (0.80), Lca^*^ and Lcb^*^ (−0.92), Ll and Lw (0.83), Hvd and Htd (0.80), Hvd and Hw (0.89), Cl and Cl/Hvd (0.94), and Htd and Hw (0.95). Meanwhile, Lx, Ln, Pl, Pw, Lm, Ls, Lmc, Dmc, and Cfc showed low correlations with other traits.

These results indicated the key traits with close and wide relationships with others were Pd, Ph, Ll, Lw, Hcb^*^, Hvd, and Htd, which deserved more attention in cabbage breeding.

### QTL analysis for cabbage main agronomic traits

**Figure 3:**
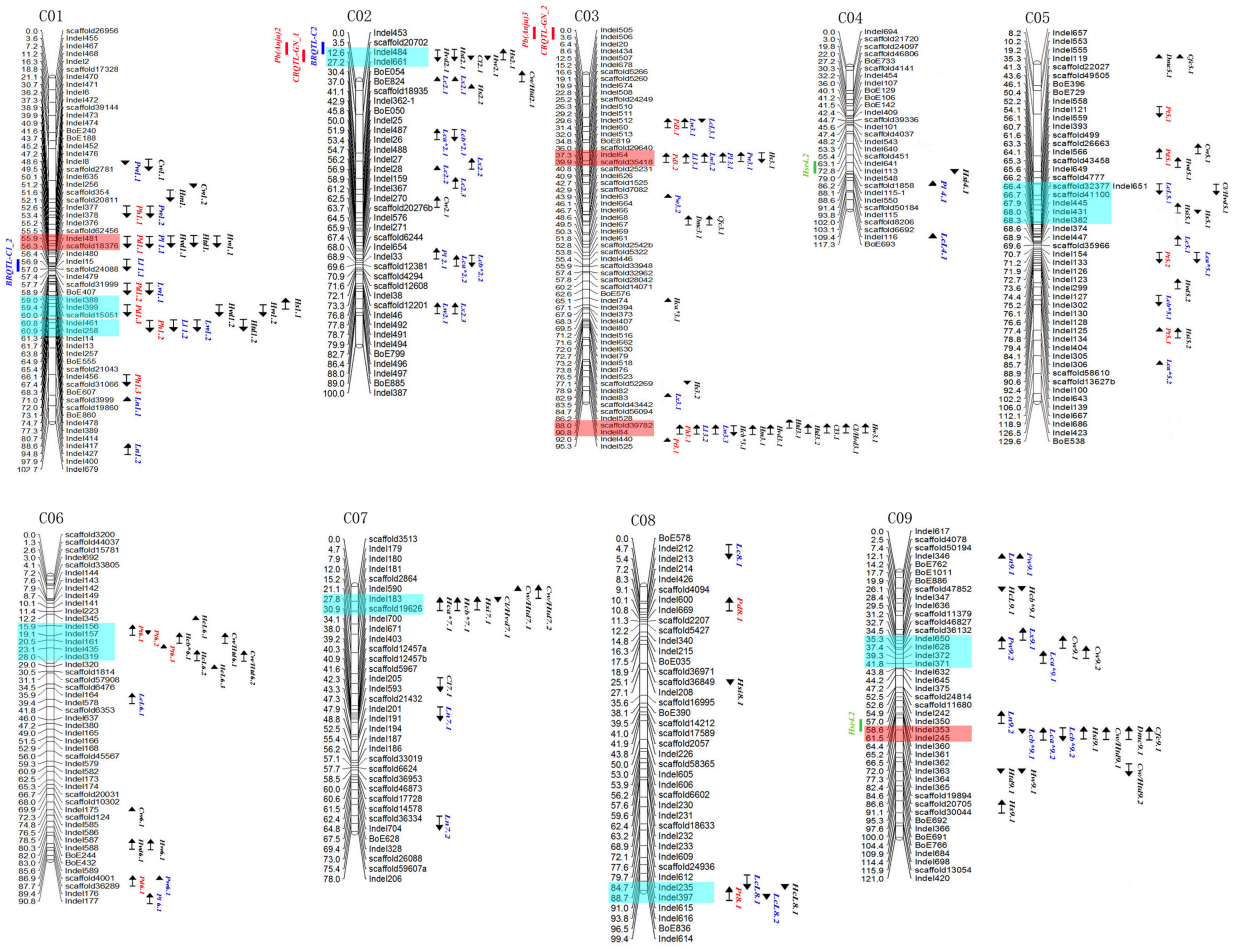
**QTL mapping for cabbage agronomic traits in genetic lingkage maps**. Marker locations are listed to the right and recombination distances (cM) to the left of each linkage group. Locations of QTLs are indicated by names, bars and arrows to the right of the linkage groups (Red, Plant type related traits; Blue, Leaf related traits; Black, head related traits). Arrows indicate the relative effect of the 96-100-308 allele with upward for increasing and downward for decreasing. Blue blocks and red ones represent QTL clusters and significant ones. QTLs from the previous studies were indicated on the left of the linkage groups (blue, black rot resistance; red, clubroot resistance; greed, head splitting resistance). For abbreviations, see Table 1.

QTL mapping results are shown in Figure 3. In total 144 QTLs with a LOD threshold of >3.0 were detected for 24 cabbage main agronomic traits. Each QTL explained 6.0–55.7% of phenotype variation. Of all the QTLs, 68.1% had a contribution rate (CR) higher than 10%, and 35.4% could be detected in more than one season.

### QTL analysis for plant-type-related traits

Twenty-five QTLs related to four plant-type-related traits were detected on chromosomes C01, C03, C05, C06, and C08 (Figure 3, indicated in red), with each explaining 6.4–55.7% of phenotype variation (Table 5). Five (total contribution rate, TCR of 14.6–22.7%), five (TCR 17.5–29.8%), nine (TCR 35.8–43.0%), and four QTLs (TCR 36–62.1%) were identified for Pt1, Pt2, Pd, and Ph, respectively. Of the QTLs, 72% had CRs higher than 10%, with 40% of these QTLs detected in more than one season.

**Table 5:** **Identification of QTLs associated with plant type related trait in cabbage**.

**Trait**	**Season**	**QTL**	**Chr^a^**	**Position (cM)^b^**	**Peak marker/marker interval^c^**	**LOD**	***R*^2^ (%)^d^**	**Add^e^**
Plant type (Pt1)	12a	*Pt 3.1*	3	92	Indel440	6.07	12.5	2.47
	12s	*Pt 5.1*	5	54.1–56.1	Indel121–559	4.7	14.6	−1.83
	12a	*Pt 5.2*	5	70.7–71.2	Indel154–133	4.95	10.2	−2.12
	11a	*Pt 6.2*	6	19.1	Indel157	3.4	8.1	−1.69
	11a	*Pt 8.1*	8	84.7–88.7	Indel235–397	4.64	11.3	2.01
Plant type (Pt2)	12s	*Pt 5.3*	5	77.4	Indel125	5.76	17.5	0.38
	**12a**	***Pt 6.1***	**6**	**15.9–19.1**	**Indel156–157**	**9.84**	**21**	**0.37**
	**11a**	***Pt 6.2***	**6**	**19.1**	**Indel157**	**3.38**	**9.1**	**0.27**
	11a	*Pt 6.3*	6	23.1	Indel435	3.38	9.1	0.27
	11a	*Pt 8.1*	8	84.7–88.7	Indel235–397	4.28	11.6	−0.3
Plant diameter	**12s**	***Pd 1.1***^f^	**1**	**55.9–56.3**	**Indel481–scaffold18376**	**4.16**	**8**	−**1.93**
	**12a**	***Pd 1.2***	**1**	**57.7–58.9**	**scaffold31999–BoE407**	**10.59**	**15.1**	−**3.16**
	**11a**	***Pd 1.3***	**1**	**59.4–60**	**Indel399–scaffold15051**	**6.79**	**17**	−**3.31**
	11a	*Pd 3.1*	3	29.6–31.4	Indel512–60	4.42	10.6	2.68
	12a	*Pd 3.2*	3	37.3–39.9	Indel64–scaffold35418	12.76	19.6	3.42
	12s	*Pd 3.2*	3	37.3–39.9	Indel64–scaffold35481	10.04	21.2	2.97
	12s	*Pd 5.1*	5	64.1–65.3	Indel566–scaffold43458	5.26	10.3	1.95
	11a	*Pd 6.1*	6	86.9–87.7	scaffold4001–36289	3.52	8.2	2.44
	12a	*Pd 8.1*	8	10.1–10.8	Indel600–699	6.23	8.3	2.08
Plant height	12a	*Ph 1.1*	1	52.6–53.4	Indel377–378	6.17	12.5	−2.24
	11a	*Ph 1.2*	1	60.8–60.9	Indel461–258	6.91	16.9	−2.2
	12s	*Ph 1.3*	1	66.1–67.4	Indel456–scaffold31066	5.2	6.4	−1.72
	**11a**	***Ph 3.1***	**3**	**88–90.8**	**scaffold39782–Indel84**	**10.39**	**27**	**2.73**
	**12a**	***Ph 3.1***	**3**	**88–90.8**	**scaffold39782–Indel84**	**14.16**	**23.5**	**2.96**
	**12s**	***Ph 3.1***	**3**	**88–90.8**	**scaffold39782–Indel84**	**28.2**	**55.7**	**4.66**

*For abbreviations, see Table 1*.

a *The chromosome number*.

b *The position of the peak marker or marker interval*.

c *Peak marker or the marker interval*.

d *The proportion of the phenotypic variance explained by each QTL*.

e *Additive effect: positive additivity indicated that 96-100-308 carries the allele for an increase in the trait value, while negative additivity means that 01-20 carries the allele for an increase in the trait value*.

f *Robust QTLs were indicated in bold*.

Robust QTLs included *Pt 6.2* and *Pt 8.1*, which could be detected through both visual and manual assay methods. *Pd 3.2* showed a positive additive effect and was detected in two seasons, with CRs of 19.6–21.2%; *Ph 3.1*, which explained 23.5–55.7% of phenotypic variation, was detected in all three seasons with LOD scores over 10.0, while positive effects indicated the locus *Ph 3.1* from parent 96-100-308 contributed to the favorable alleles.

Important clusters associated with plant-type-related traits included: the 15.9–19.1 cM region (Indel156–157, for Pt) on chromosome C06; 57.7–60.0 cM region (scaffold31999–scaffold15051, for Pd) on chromosome C01, and the 37.3–39.9 cM (Indel64–scaffold35418, for Pd) and 88.0–90.8 cM regions (scaffold39782–Indel84, for Ph) on chromosome C03.

### QTL analysis for leaf-related traits

Sixty-four QTLs for 10 leaf-related traits were detected on all nine chromosomes (Figure 3, indicated in blue), with each QTL explaining 6.0–31.7% of phenotypic variation. Of the QTLs, 67.2% had CRs higher than 10% (Table 6); 39.1% of these QTLs could be detected in more than one season.

**Table 6:** **Identification of QTLs associated with cabbage leaf traits using a DH population in three seasons**.

**Trait**	**Season**	**QTL**	**Chr**	**Position (cM)**	**Peak marker/marker interval**	**LOD**	***R*^2^ (%)**	**Add**
Leaf Color (Visual measurement)	12a	*Lc 2.1*	2	37	BoE824	6.02	15.6	0.35
	11a	*Lc 2.2*	2	56.9	Indel28	3.3	10.8	0.34
	12s	*Lc 2.3*	2	58.9–61.2	Indel159–367	4.07	11.6	0.33
	12a	*Lc 5.1*	5	68.9–69.6	Indel447–scaffold35966	5.59	12.5	0.38
	12s	*Lc 8.1*	8	4.7–5.4	Indel212–213	3.71	10.3	−0.3
Leaf Color a^*^	12a	Lca^*^ 2.1	2	51.9–53.4	Indel487–26	4.6	8.5	0.4
	12s	Lca^*^ 2.2	2	68.9–69.6	Indel33–scaffold12381	6.27	12.9	0.41
	**12s**	Lca^*^5.1	**5**	**69.6–70.7**	**Indel154–133**	**8.14**	**17.4**	**0.42**
	**11a**	Lca^*^5.1	**5**	**70.7**	**Indel154**	**6.06**	**14.4**	−**0.45**
	12a	Lca^*^ 5.2	5	85.7	Indel306	4.06	7.5	0.39
	12a	Lca^*^ 9.1	9	39.3–41.8	Indel372–371	5.76	10.5	0.45
	**11a**	Lca^*^9.2	**9**	**58.6–61.5**	**Indel353–245**	**4.09**	**9.5**	−**0.35**
	**12s**	Lca^*^9.2	**9**	**58.6–61.5**	**Indel353–245**	**4.49**	**8**	−**0.34**
Leaf Color b^*^	12a	Lcb^*^ 2.1	2	51.9–53.4	Indel487–26	5.98	12.3	−1.08
	12s	Lcb^*^ 2.2	2	68.9–69.6	Indel33–scaffold12381	8.82	18.5	−1.03
	**11a**	Lcb^*^5.1	**5**	**74.4–75.2**	**Indel127–302**	**5.81**	**12.3**	−**0.72**
	**12s**	Lcb^*^5.1	**5**	**74.4–75.2**	**Indel127–302**	**7.12**	**14.5**	−**0.95**
	**12a**	Lcb^*^9.1	**9**	**58.6**	**Indel353**	**4.75**	**9.4**	−**0.94**
	**11a**	Lcb^*^9.2	**9**	**58.6–61.5**	**Indel353–245**	**4.03**	**9.5**	−**0.57**
	**12s**	Lcb^*^9.2	**9**	**58.6–61.5**	**Indel353–245**	**4.14**	**8**	−**0.67**
Leaf Color L	12s	*LcL 3.1*	3	29.6	Indel512	4.34	11.3	−0.81
	12a	*LcL 4.1*	4	109.4	Indel116	4.19	7.8	0.49
	12a	*LcL 5.1*	5	66.4–66.7	Indel651–scaffold41100	4.34	8.1	−0.52
	11a	*LcL 6.1*	6	35.9–39.4	Indel164–578	5.67	11.4	0.72
	**11a**	***LcL 8.1***	**8**	**79.7–84.7**	**Indel612–235**	**11.64**	**26.1**	−**1.04**
	**12s**	***LcL 8.1***	**8**	**84.7**	**Indel235**	**5.23**	**13.9**	−**0.81**
	12a	*LcL 8.2*	8	88.7	Indel397	3.46	6.4	−0.46
Leaf wax powers	12a	*Lx 2.1*	2	37	BoE824	3.75	17.9	0.43
	11a	*Lx 2.2*	2	56.2–56.9	Indel27–28	4.45	10.1	0.41
	**12a**	***Lx 2.3***	**2**	**73.3–76.8**	**scaffold12201–Indel46**	**6.76**	**13.7**	**0.38**
	**12s**	***Lx 2.3***	**2**	**76.8**	**Indel46**	**5.75**	**17.5**	**0.47**
	11a	*Lx 3.1*	3	78.9	Indel82	4.43	10	0.4
	**12a**	***Lx 9.1***	**9**	**34.5–35.3**	**scaffold36132–Indel650**	**4.5**	**8.9**	**0.3**
	**11a**	***Lx 9.1***	**9**	**34.5–35.3**	**scaffold36132–Indel650**	**8.01**	**19.2**	**0.55**
Leaf number	12a	*Ln 1.1*	1	71	scaffold3999	3.51	6.6	0.97
	12s	*Ln 1.2*	1	88.6–94.8	Indel417–427	5.48	16.8	1.86
	12a	*Ln 7.1*	7	47.9–48.8	Indel201–191	4.98	9.6	−1.06
	11a	*Ln 7.2*	7	62.4–64.8	scaffold36334–Indel704	5.42	15	−1.09
	12a	*Ln 9.1*	9	12.1	Indel346	3.57	6.7	0.89
	11a	*Ln 9.2*	9	54.9–57	Indel242–350	4.47	11.9	0.95
Leaf length	12a	*Ll 1.1*	1	56.9–57	Indel15–scaffold24088	11.21	19.6	−2.27
	**11a**	***Ll 1.2***	**1**	**60.8–60.9**	**Indel461–258**	**8.45**	**21.9**	−**2.22**
	**12s**	***Ll 1.2***	**1**	**60.8–60.9**	**Indel461–258**	**8.12**	**15.9**	−**1.6**
	**12a**	***Ll 3.1***	**3**	**37.3–39.9**	**Indel64–scaffold35418**	**8.74**	**14.9**	−**1.88**
	**11a**	***Ll 3.1***	**3**	**37.3–39.9**	**Indel64**	**3.13**	**6.4**	**1.29**
	**11a**	***Ll 3.2***	**3**	**88–90.8**	**scaffold39782–Indel84**	**7.34**	**18.7**	**2**
	**12s**	***Ll 3.2***	**3**	**88–90.8**	**scaffold39782–Indel84**	**13.95**	**31.7**	**2.13**
Leaf width	12a	*Lw 1.1*	1	57.7–58.9	scaffold31999–BoE407	9.17	18.1	−1.77
	11a	*Lw 1.2*	1	60.8–60.9	Indel461–258	8.84	24	−2.08
	12s	*Lw 2.1*	2	73.3–76.8	scaffold12201–Indel46	5.29	12.7	1.2
	12a	*Lw 3.1*	3	29.6–31.4	Indel512–60	3.31	6	0.98
	11a	*Lw 3.2*	3	37.3–39.9	Indel64–scaffold35418	5.03	12.7	1.48
	12s	*Lw 3.3*	3	88–90.8	scaffold39782–Indel84	6.19	16	1.39
Petiole length	12a	*Pl 2.1*	2	68–68.9	Indel33–scaffold12381	6.25	10.2	1.07
	12a	*Pl 3.1*	3	37.3–39.9	Indel64–scaffold35418	5.93	9.6	1.12
	12s	*Pl 4.1*	4	86.2	scaffold1858	3.05	10	0.83
	12a	*Pl 6.1*	6	89.4–90.8	Indel176–177	6.7	11.1	1.26
Petiole width	12s	*Pw 1.1*	1	48.6	Indel8	4.7	10.6	−0.13
	12a	*Pw 1.2*	1	52.6–53.4	Indel377–378	6.07	9.7	−0.13
	12a	*Pw 3.1*	3	37.3–39.9	Indel64–scaffold35418	4.4	7	0.11
	12a	*Pw 3.2*	3	43.9	Indel63	5.68	13	0.13
	12a	*Pw 6.1*	6	86.9	scaffold4001	3.92	6.3	0.11
	12s	*Pw 9.1*	9	12.1	Indel346	3.78	8.4	0.1
	12a	*Pw 9.2*	9	35.3–37.4	Indel650–628	4.48	6.9	0.1

*Robust QTLs were indicated in bold*.

The QTLs identified were: five (TCR 10.8–28.1%) for Lc, eight (TCR 23.9–38.3%) for Lca^*^, nine (TCR 35.8–43%) for Lcb^*^, seven (TCR 22.3–37.5%) for LcL, seven (TCR 17.5–40.5%) for Lx, Six (TCR 16.8–26.9%) for Ln, seven (TCR 34.5–47.6%) for Ll, six (TCR 24.1–36.7%) for Lw, four (TCR 10–30.9%) for Pl, and seven (TCR 19–42.9%) for Pw.

Robust QTLs or regions included: a 4-Mb region on chromosome C08 containing *LcL 8.1* and *LcL 8.2*, detectable in all three seasons, with maximum contribute rate (CR) and LOD of 26.1 and 11.6, respectively; *Lx 2.3* and *Lx 9.1* were detected in two seasons, with *Lx 9.1* having a max CR of 19.2%; *Ll 1.2, Ll 3.1*, and *Ll 3.2* could be detected in more than one season, with *Ll 3.2* having a max CR of 31.7; and the region containing *Lw 1.1* and *Lw 1.2* was detected in two seasons, with a max CR of 24%.

We also found the same QTLs for different traits, indicating that they might be controlled by common genetic factors. For example, the QTLs were almost the same for Lca^*^ and Lcb^*^ and 14 out of 15 could be detected in more than one season, and this situation was also similar for Ll and Lw QTLs.

The results revealed important genetic factors associated with leaf-related traits were mainly located on chromosomes C02, C03, and C09 (Figure 3). Important regions included: chromosome C02, 51.9–56.9 cM (Indel487–28, for Lc and Lx) and 68.0–69.6 cM (Indel654–scaffold12381, for Lc and Pl); chromosome C03, 37.3–39.9 cM (Indel64–scaffold35418, for Pw, Pl, Lw, and Ll), and 88.0–90.8 cM (scaffold39782–Indel84, for Lw and Ll); and chromosome C09, 12.1–14.2 cM (Indel346–BoE762, for Ls, Pw, and Ln) and 58.6–61.5 cM (Indel353–Indel245, for Lc).

### QTL analysis for head-related traits

Fifty-five QTLs for head-related traits were detected on nine chromosomes (Figure 3, indicated in black), with each explaining 6.0–28.5% of phenotypic variation. Of the QTLs, 67.3% had CRs higher than 10% (Table 7), and 29.1% of these were detected in more than one season.

**Table 7:** **Identification of QTLs associated with cabbage head traits using a DH population in three seasons**.

**Trait**	**Season**	**QTL**	**Chr**	**Position (cM)**	**Peak marker/marker interval**	**LOD**	***R*^2^ (%)**	**Add**
Head color a^*^	12s	Hca^*^ 3.1	3	65.1	Indel74	3.37	10.6	0.41
	11a	Hca^*^ 7.1	7	27.8–30.9	Indel183–scaffold19626	5.78	18.9	0.57
Head color b^*^	12s	Hcb^*^ 3.1	3	88-90.8	scaffold39782–Indel84	4.97	12.9	−0.99
	12s	Hcb^*^ 6.1	6	19.1-20.5	Indel157–161	5.73	15	1.01
	11a	Hcb^*^ 7.1	7	27.8–30.9	Indel183–scaffold19626	5.85	19.2	1.55
	12a	Hcb^*^ 9.1	9	26.1	scaffold47852	3.84	8.1	−0.79
Head color L	11a	*HcL 6.1*	6	23.1	Indel435	3.95	15	1.74
	12s	*HcL 6.2*	6	23.1–28	Indel435–319	7.02	20.9	1.42
	12a	*HcL 6.3*	6	30.5	scaffold1814	3.27	6.4	0.65
	12a	*HcL 8.1*	8	84.7	Indel235	3.03	6	−0.62
	12a	*HcL 9.1*	9	26.1	scaffold47852	4.35	9.3	−0.72
Head transverse diameter	12a	*Htd 1.1*	1	55.9–56.3	Indel481–scaffold18376	6.11	11.2	−0.71
	11a	*Htd 1.2*	1	60.8–60.9	Indel461–258	5.32	13.9	−0.97
	**12a**	***Htd 3.1***	**3**	**86.2–88**	**Inde528–scaffold39782**	**4.91**	**8.9**	**0.59**
	**11a**	***Htd 3.2***	**3**	**88–90.8**	**scaffold39782–Indel84**	**3.87**	**10**	**0.81**
	**12s**	***Htd 3.2***	**3**	**88–90.8**	**scaffold39782–Indel84**	**9.49**	**28.5**	**0.72**
	**11a**	***Htd 9.1***	**9**	**72**	**Indel363**	**3.34**	**8.3**	−**0.7**
	**12a**	***Htd 9.1***	**9**	**72**	**Indel363**	**4.48**	**8.2**	−**0.54**
Core width	12a	*Cw 1.1*	1	48.6–49.5	Indel8–scaffold2781	8.33	16.1	−0.15
	11a	*Cw 1.2*	1	51.2	Indel256	5.58	14.9	−0.15
	12s	*Cw 2.1*	2	62.5–63.7	Indel270–scaffold20276b	8.09	15.7	0.16
	12s	*Cw 5.1*	5	63.3–64.1	scaffold26663–Indel566	8.22	16	0.17
	12s	*Cw 6.1*	6	69.9	Indel175	3.65	6.4	0.13
	**11a**	***Cw 9.1***	**9**	**35.3–37.4**	**Indel650–628**	**5.77**	**15.5**	**0.13**
	**12a**	***Cw 9.2***	**9**	**37.4–39.3**	**Indel628–372**	**4.8**	**7.6**	**0.1**
Core width/Head transverse diameter	12a	*Cw/Htd 2.1*	2	30.4–37	BoE054–824	6.63	11.5	0.01
	12s	*Cw/Htd 6.1*	6	19.1–20.5	Indel157–161	6.77	14.2	0.02
	12a	*Cw/Htd 6.2*	6	23.1–28	Indel435–319	5.48	8.3	0.01
	11a	*Cw/Htd 7.1*	7	21.1	Indel590	4.69	11	0.01
	**12a**	***Cw/Htd 7.2***	**7**	**27.8**	**Indel590–183**	**4.97**	**8.2**	**0.01**
	**12s**	***Cw/Htd 7.2***	**7**	**21.1–27.8**	**Indel590–183**	**6.46**	**13.7**	**0.02**
	12s	*Cw/Htd 9.1*	9	58.6–61.5	Indel353–245	6.34	13.2	0.01
	**12a**	***Cw/Htd 9.2***	**9**	**66.5–72**	**Indel362–363**	**10.38**	**17**	**0.01**
	**11a**	***Cw/Htd 9.2***	**9**	**66.5–72**	**Indel362–363**	**10.25**	**26.6**	**0.01**
Head shape index	12a	*Hsi 2.1*	2	12.6–27.2	Indel484–661	8.59	14.2	−0.05
	12s	*Hsi 4.1*	4	72.8	Indel113	4.55	12	−0.03
	12a	*Hsi 5.1*	5	67.9–68	Indel445–431	10.41	16.4	0.05
	11a	*Hsi 5.2*	5	77.4–78.8	Indel125–134	10.19	27.8	0.09
	12a	*Hsi 7.1*	7	27.8–30.9	Indel183–scaffold19626	4.75	7	0.04
	12s	*Hsi 8.1*	8	25.1	scaffold36849	4.52	11	−0.03
	12s	*Hsi 9.1*	9	58.6–61.5	Indel353–245	3.96	9.6	0.03
Head Solidity	11a	*Hs 1.1*	1	59–59.4	Indel388–399	3.89	8.9	0.04
	**12a**	***Hs 2.1***	**2**	**12.6–27.2**	**Indel484–661**	**7.19**	**13.9**	**0.04**
	**11a**	***Hs 2.1***	**2**	**27.2**	**Indel661**	**4.27**	**11.6**	**0.04**
	12s	*Hs 2.2*	2	41.1	scaffold18935	6.86	20.5	0.03
	12a	*Hs 3.1*	3	37.3–39.9	Indel64–scaffold35418	4.92	8.7	−0.04
	11a	*Hs 3.2*	3	76.5	Indel523	3.11	7.2	−0.03
	11a	*Hs 5.1*	5	68	Indel431	3.32	7.6	−0.03
	12a	*Hs 9.1*	9	86.6–91.1	scaffold20705–30044	5.3	9.3	0.03
Dry matter cotent	12s	*Dmc 3.1*	3	48.6–49.5	Indel68–67	6.11	14.4	0.28
	12s	*Dmc 5.1*	5	35.3	Indel119	6.7	17.8	0.3
	12s	*Dmc 9.1*	9	58.6–61.5	Indel353–245	4.4	10.1	0.22
Crude fiber content	12s	*Cfc 3.1*	3	48.6–49.5	Indel68–67	6.49	13.9	0.03
	12s	*Cfc 5.1*	5	35.3	Indel119	7.38	17.7	0.03
	12s	*Cfc 9.1*	9	58.6–61.5	Indel353–245	6.48	13.9	0.03

*Robust QTLs were indicated in bold*.

QTLs identified consisted of: 11 (TCR 8.1–21.7%) for head color coordinates a^*^, b^*^, and L; seven (TCR 28.3–32.2%) for Htd, seven (TCR 23.7–38.1%) for Cw, nine (TCR 37.6–45%) for Cw/Htd, seven (TCR 27.8–37.6%) for Hsi, and eight (TCR 20.5–35.3%) for Hs.

Robust QTLs or regions included: Hcb^*^ *7.1*, detected for both a^*^ and b^*^, with CRs of over 18%; Hcb^*^ *9.1*, detected for both b^*^ and L; the region (Indel528–Indel84) containing *Htd 3.1* and *Htd 3.2*, with a maximum CR of 28.5%, which could be detected in all three seasons; *Cw/Htd 9.2*, which explained 17.0–26.6% of phenotypic variance over two seasons; and the allele from 96 to 100, which increased Cw/Htd in three seasons, explained 27.8% of phenotypic variance. Other QTLs detected in more than one season included *Htd 9.1, Hsi 5.2, Hs 2.1*, and two regions: Indel8–Indel236 containing *Cw 1.1* and *Cw 1.2*, and Indel650–Indel372 containing *Cw 9.1* and *Cw 9.2*. The QTLs identified for Dmc and Cfc were identical, consistent with the fact that Cfc was the main content of Dmc. Our previous study identified QTLs related to cabbage heading traits, including Hm, Hw, Cl, Hvd, and Cl/Hvd (Lv et al., 2014); these were also indicated on the chromosomal diagram to provide more comprehensive information (Figure 3).

The results indicated important QTL clusters associated with head-related traits were mainly located on chromosomes C01, C02, C03, C05, C07, and C09 (Figure 3). Significant regions included: chromosome C01, 55.9–56.3 cM region (Indel481–scaffold18376, for Hw, Hvd, and Htd) and 59.0–60.9 cM region (Indel388–Indel258, for Hsi, Hw, Htd, and Hvd); chromosome C02, 12.6–27.2 cM region (Indel484–Indel661, for Hs, Hw, Cl, Hsi, and Hvd); chromosome C03, 88.0–90.8 cM region (scaffold39782–Indel84, for Hw, Cl/Hvd, Cl, Htd, Hvd, Hm, and Hc); chromosome C05, 63.3–68.0 cM region (scaffold26663–Indel341, for Cw, Cl/Hvd, Hvd, Hs, and Hsi); chromosome C07, 27.8–30.9 cM region (Indel183–scaffold19626, for Cw/Htd, Cl/Hvd, his, and Hc); and chromosome C09, 58.6–61.5 cM region (Indel353–Indel245, for Cw/Htd, Hsi, Cfc, and Dmc).

### QTL clusters detection revealed significant genomic regions

To identify significant genomic regions harboring several QTLs associated with important agronomic traits, we indicated positions of all the QTLs on the chromosomes (Figure 3). Twelve QTL clusters, i.e., hot regions, were detected on all chromosomes except for chromosome C04. The clusters were listed in Table 8 in accordance with the reference genome of cabbage on BRAD. The most significant four clusters were indicated in red, including Indel481–scaffold18376 (3.20 Mb) on C01, with five QTLs for five traits, Indel64–scaffold35418 (2.22 Mb) on C03, with six QTLs for six traits, scaffold39782–Indel84 (1.78 Mb) on C03, with 10 QTLs for 10 traits, and Indel353–Indel245 (9.89 Mb) on C09, with seven QTLs for six traits.

**Table 8:** **Analysis of QTL clustering regions**.

**Marker interval**	**Physical position**	**Chr**.	**QTL**	**CR (%)**
**Indel481–scaffold18376; 55.9–56.3 cM**^a^	**21,380,421–24,540,688; 3.20 Mb**	**1**	***Hw1.1, Htd1.1, Hvd1.1, Pl1.1, Pd1.1***	**s**
Indel388–scaffold15051; 59.0–60.0 cM	–	1	*Hs1.1, Hw1.2, Hvd1.2, Pd1.3*	–
Indel461-Indel258; 60.8–60.9 cM	–	1	*Htd1.2, Lw1.2, Ll1.2, Ph1.2*	–
Indel484-Indel661; 12.6–27.2 cM	–	2	*Hs2.1, Hw2.1, Cl2.1, Hsi2.1, Hvd2.1*	–
**Indel64–scaffold35418; 37.3–39.9 cM**	**16,518,329–18,734,806; 2.22 Mb**	**3**	***Hs3.1, Pw3.1, Pl3.1, Lw3.1, Ll3.1, Pd3.2***	**7–21.2**
**Scaffold39782-Indel84; 88.0–90.8 cM;**	**46,066,158–47,848,803; 1.78 Mb**	**3**	Hw3.1, Cl/Hvd3.1, Cl3.1, Htd3.2, Hvd3.1, Hm3.1, Hcb^*^3.1, Ls3.1, Lw3.3, Ll3.2, Ph3.1,	**12.9–55.7**
Scaffold32377–Indel382; 66.4–68.3 cM	–	5	*Cl/Hvd5.1, Hs5.1, Hsi5.1, Lmc5.1, LcL5.1*	–
Indel156–Indel319; 15.9–28 cM	–	6	Cw/Htd6.2, Cw/Htd6.1, HcL6.2, Hcb^*^6.1, Pt6.3, Pt6.1, Pt 6.2	–
Indel183–scaffold19626; 27.8–30.9 cM	–	7	Cl/Hvd7.1, Hsi7.1, Hcb^*^7.1, Hca^*^7.1	–
Indel235–Indel397; 84.7–88.7 cM	–	8	*HcL8.1, LcL8.2, LcL8.1, Pt8.1*	–
Indel650–Indel371; 35.3–41.8 cM	–	9	Cw9.1, Cw9.2, Hca^*^9.1, Lx9.1, Pw9.2	–
**Indel353-Indel245; 58.6–61.5 cM**	**11,545,910–21,416,629; 9.87 Mb**	**9**	Cfc9.1, Dmc9.1, Cw/Htd9.1, Hsi9.1, Lcb^*^9.2, Lcb^*^9.1, Lca^*^9.1	**9.4–13.9**

*For abbreviations, see Table 1*.

a *The most significant regions were indicated in bold*.

Except for the QTLs for 24 main agronomic traits in the current study, QTL positions from previous studies were also added according to their flanking marker positions (Figure 3). These important QTLs include black rot resistance (*BRQTL-C1_2* and *BRQTL-C2*) (Kifuji et al., 2013; Lee et al., 2015), head splitting resistance (*Hsr4.2* and *Hsr9.2*) (Su et al., 2015) and clubroot resistance [*Pb(Anju)2, Pb(Anju)3, CRQTL-GN_1*, and *CRQTL-GN_2*], (Nagaoka et al., 2010; Lee et al., 2016). Results showed that the black rot resistance QTL *BRQTL-C2*, and clubroot resistance *CRQTL-GN_1*and *Pb(Anju)2* were located in the cluster on chromosome C02 containing *Hs2.1, Hw2.1, Cl2.1, Hsi2.1, Hvd2.1*; the head splitting resistance QTL *Hsr9.2* was located near the cluster on C09 containing Cfc9.1, Dmc9.1, Cw/Htd9.1, Hsi9.1, Lcb^*^9.2, Lcb^*^9.1, Lca^*^9.1. These results indicated that these traits were possibly controlled by common genic factors on the corresponding genomic regions.

Meta-QTL analysis was conducted to further confirm the positions of the QTL clusters. Results showed that the positions of the meta-QTLs compromising more than five QTLs were almost the same with the QTL clusters (Supplementary Figure 2).

### Candidate genes analysis for the major QTLs and QTL cluster regions

The candidate genes for seven major QTLs or QTL clusters (meta-QTLs) were analyzed based on the annotations for the *B. oleracea* reference genome acquired from BRAD (for gene search result, see Supplementary Table 1). The annotation included transcription factor, proteolysis, ATP binding, tRNA methylation, kinase, protein phosphorylation, transmembrane transport, etc. Some of the genes might be good candidates associated with related traits according to the alignment results with Arabidopsis, especially those related to hormonal pathways (e.g., Bol036563), transcriptions factors (e.g., Bol021949) and photosystem components (e.g., Bol Bol013750).

## Discussion

### QTL analysis of main agronomic traits on cabbage

In recent years, as diseases like black rot are aggregating and new diseases like clubroot are emerging, researchers have paid more attention to QTLs related to resistance, such as the resistance to *S. sclerotiorum* (Mei et al., 2013), black rot resistance (Kifuji et al., 2013; Lee et al., 2015), clubroot resistance (Lee et al., 2016), and resistance to Diamondback moth (Ramchiary et al., 2015). However, the breeder and the growers also care greatly about other important agronomic traits, such as heading and quality traits. Here, for the first time, we report a comprehensive QTL analysis of the main cabbage agronomic traits using a cabbage DH population. In total, 144 QTLs with LOD thresholds of >3.0 were detected for 24 traits. We identified major QTLs and important QTL clusters associated with these traits. These QTLs will be helpful in the identification of genes related to these traits, and to facilitate MAS for cabbage breeders.

Many factors could affect the QTL detection efficiency, and the main ways to improve it include enlarging population size, increasing the number of markers and performing precise phenotype measurement (Li et al., 2010). In the current study, for example, Ls, Lm, and Lmc showed almost no difference in parental lines and irregular segregation pattern, which was likely caused by inaccurate phenotype measurement. This was proved in the mapping analysis: no major QTL was detected for them (data not shown). However, normal distribution was not a necessity for QTL detection: the trait values fitted to the normal distribution only under the polygenic hypothesis; in other cases, they did not fit the normal distribution when the number of QTLs was few and the CR was high (Lynch and Walsh, 1998; Zhai and Wang, 2007). The current study used an intra-subspecies heading cabbage DH population with 196 lines originating from two elite parental lines 01-20 and 96-100-308, and applied agronomic trait assays in three seasons. This could help to reduce errors and to improve the accuracy and precision of QTL detection.

### QTL clusters provide evidence for associated traits selection

The co-localization of QTLs was in accordance with the fact that most of them were significantly correlated with each other. And this might be caused by one or several important genes participating in more than one pathways. For example, the genes related to hormonal pathways and transcriptions factors might contribute to various biological process. The clustering of QTLs for different traits widely exists in crops. For example, the loci *Xgwm212* of “Lovrin No. 10,” a founder wheat parental line, is associated with traits of biomass, tillering, and phosphorus absorption and utilization (Zhang et al., 2006).

In the current study, 12 QTL clusters were detected on all chromosomes except for C04 (Table 8). The most significant region, i.e., scaffold39782–Indel84, was a 1.78-Mb genomic region harboring 173 genes on C03, with most of these genes having unknown or predicted functions (data not shown). Nonetheless, the QTLs and hot regions obtained in this study should prove useful for MAS in cabbage breeding programs and pave the way for further understanding of the genetic control of these traits. Besides, some of the QTLs from other previous research were also located on these clusters, such as the QTLs related to head splitting resistance and clubroot resistance, suggesting the potential probability of common genic factors for these traits, and also showing the necessity to promote further study for these regions.

The QTL clusters could also provide a molecular basis for the selection of associated traits. The QTLs in the same region usually significantly correlated with each other. For example, the Indel353–Indel245 region contained Cfc 9.1, Dmc 9.1, Lca^*^ *9.2*, and Lcb^*^ *9.2*, and the correlation analysis indicated the high Cr for Cfc and Dmc were with leaf color traits including Lc, Lca^*^ and Lcb^*^ (Table 4). This is in accordance with our experience that lighter color leaves always signify low Cfc content and crisp taste, and also suggests that it is possible to select cabbage quality traits according to leaf color in cabbage breeding. The relationships of different traits was also proved in the correlation test. Another example is, for commodity traits, head-related traits such as Hvd, Cl/Hvd, Htd, Hm, and Hw are especially important, and correlation analysis indicated Pd, Ph, Ll, and Lw were closely associated with Hvd, Htd, Hw, and Hm, suggesting their common genetic control and implying that these traits can be used to aid the selection of other important traits in cabbage breeding programs.

According to our previous study (Wang et al., 2013), the number of derived inbred lines and generated cultivars for the founder parent 01-20 reached 14 and 27, respectively. Of the 27 generated cultivars, “Zhonggan No. 21,” an early-maturing spring cabbage cultivar, has reached over 300,000 ha for the cumulative harvesting area from 2006 to 2015 in China. So what makes 01-20 a founder parental line? The answer might lie in regions like scaffold39782–Indel84 containing significant genes associated with the excellent traits including early-maturing, high production, green and round head, etc.

### Candidate genes analysis provided insights into the QTL clusters

The candidate genes for seven major QTLs or cluster regions were analyzed (Supplementary Table 1), and some of them might be good candidates associated with related traits according to the alignment results with Arabidopsis. For example, in region 2 associated with Hw, Htd, Hvd, Pl, and Pd, the homologous gene *LHCA3* in Arabidopsis is a subunit of the photosystem I antenna system (Castelletti et al., 2003) and *ARF1*, i.e., auxin response factor 1, can bind to auxin response elements and regulates plant physiology (Ulmasov et al., 1997); in region 3 associated with Hs, Pw, Pl, Lw, Ll, and Pd, the homologous gene *CIP1* interacts with *COP1*, who functions as an E3 ubiquitin ligase and mediates a variety of developmental processes in Arabidopsis (Mstsui et al., 1995; Wei and Deng, 1996) in region 4 associated with Hw, Cl/Hvd, Cl, Htd, Hvd, Hm, Hcb^*^, Lw, Ll, and Ph, the homologous gene *PXA1* is essential for photosystem II efficiency and accumulation of free fatty acids (Kunz et al., 2009); in region 7 associated with Cw/Htd and Hw, the homologous gene *PIN5* encodes a functional auxin transporter that is required for auxin-mediated development (Mravec et al., 2009). These genes might be potential candidates associated with related traits. However, the fine mapping and cloning of QTL-associated genes, especially for robust QTLs such as *Ph3.1, Ll 3.2*, and *Htd 3.2*, will require a large F_2_ population and more markers.

## Conclusions

We mapped 144 QTLs for 24 agronomic traits of heading cabbage. We also discovered 12 QTL clusters on eight chromosomes. Robust QTLs and their clusters obtained in this study should be helpful for MAS in cabbage breeding and in furthering our understanding of the genetic control of these traits.

## Author contributions

HL developed the DH populations and wrote and revised the manuscript. HL, QW isolated the samples and performed the trait and marker assays. QW, XL, and FH analyzed the trait and marker data. YZ, ZF conceived the idea and critically reviewed the manuscript. LY, MZ, YL, and ZL coordinated and designed the study. All the authors have read and approved the final manuscript.

## Funding

This work was financially supported by grants from the Key Projects in the National Science & Technology Pillar Program during the Thirteenth Five-Year Plan Period (2016YFD0100300), the Major State Basic Research Development Program (973 Program, 2012CB113906), the National Natural Science Foundation of China (31272180), the Key Projects in the National Science and Technology Pillar Program during the Twelfth Five-Year Plan Period (2012BAD02B01, 2013BAD01B04-4), the Science and Technology Innovation Program of the Chinese Academy of Agricultural Sciences (CAAS-ASTIP-IVFCAAS), and the earmarked fund for the Modern Agro-Industry Technology Research System, China (nycytx-35-gw01).

### Conflict of interest statement

The authors declare that the research was conducted in the absence of any commercial or financial relationships that could be construed as a potential conflict of interest.

## Supplementary material

Supplementary Figure 1 **Histograms for the main agronomic traits of the DH population**.

Supplementary Figure 2 **Meta-QTL analysis for the obtained 144 QTLs**.

Supplementary Table 1 **Candidate gene analysis for the eight major QTL clustering regions**.
