# Supplementary figures and images for: Whole-Genome Mapping Reveals Novel QTL Clusters Associated with Main Agronomic Traits of Cabbage (Brassica oleracea var. capitata L.)
Source: Front Plant Sci. 2016 Jul 6;7:989. doi: 10.3389/fpls.2016.00989 (PMC4933720; doi:10.3389/fpls.2016.00989)

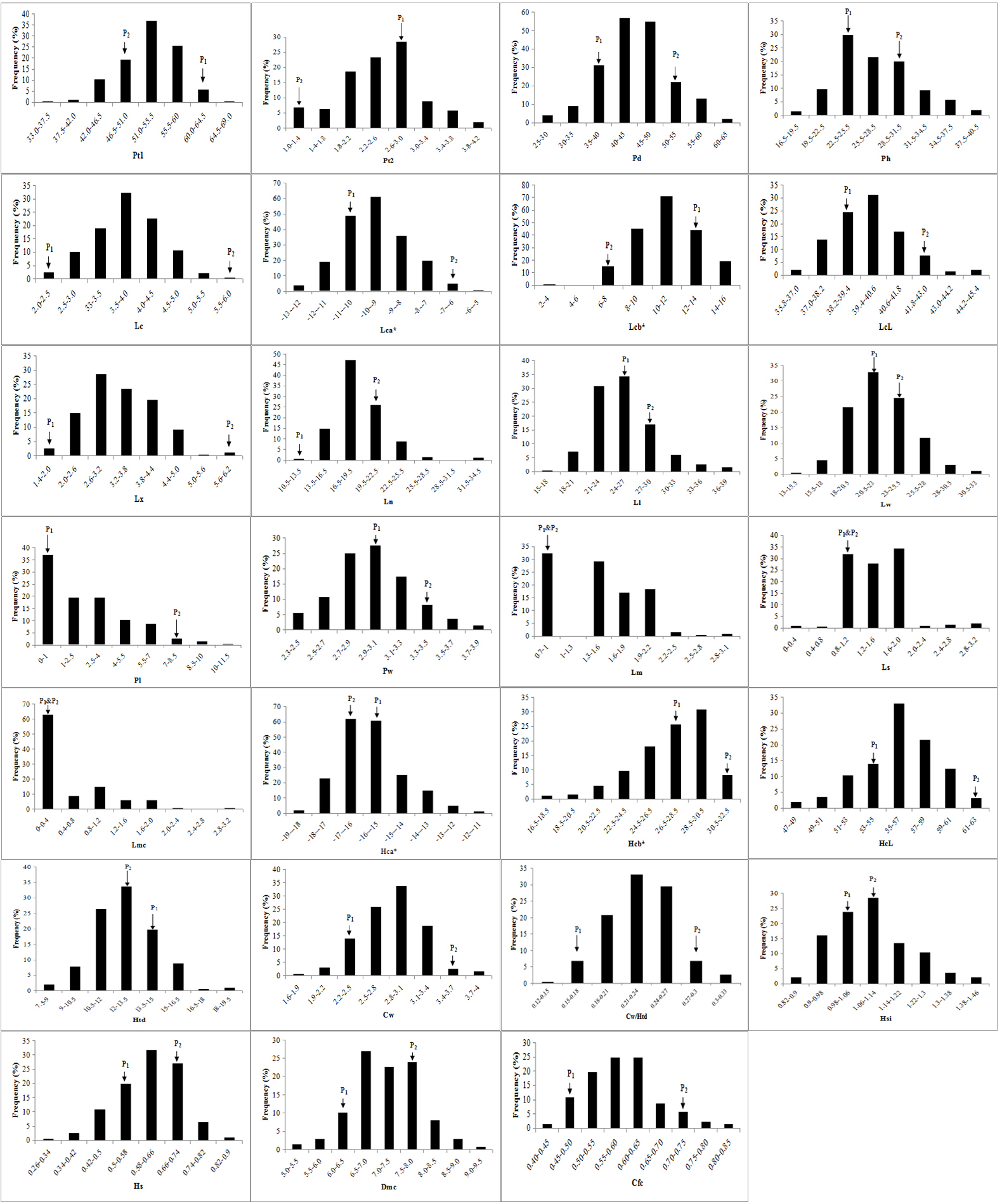

Supplement: Supplementary Figure 1 — Histograms for the main agronomic traits of the DH population. [file Image1.JPEG]

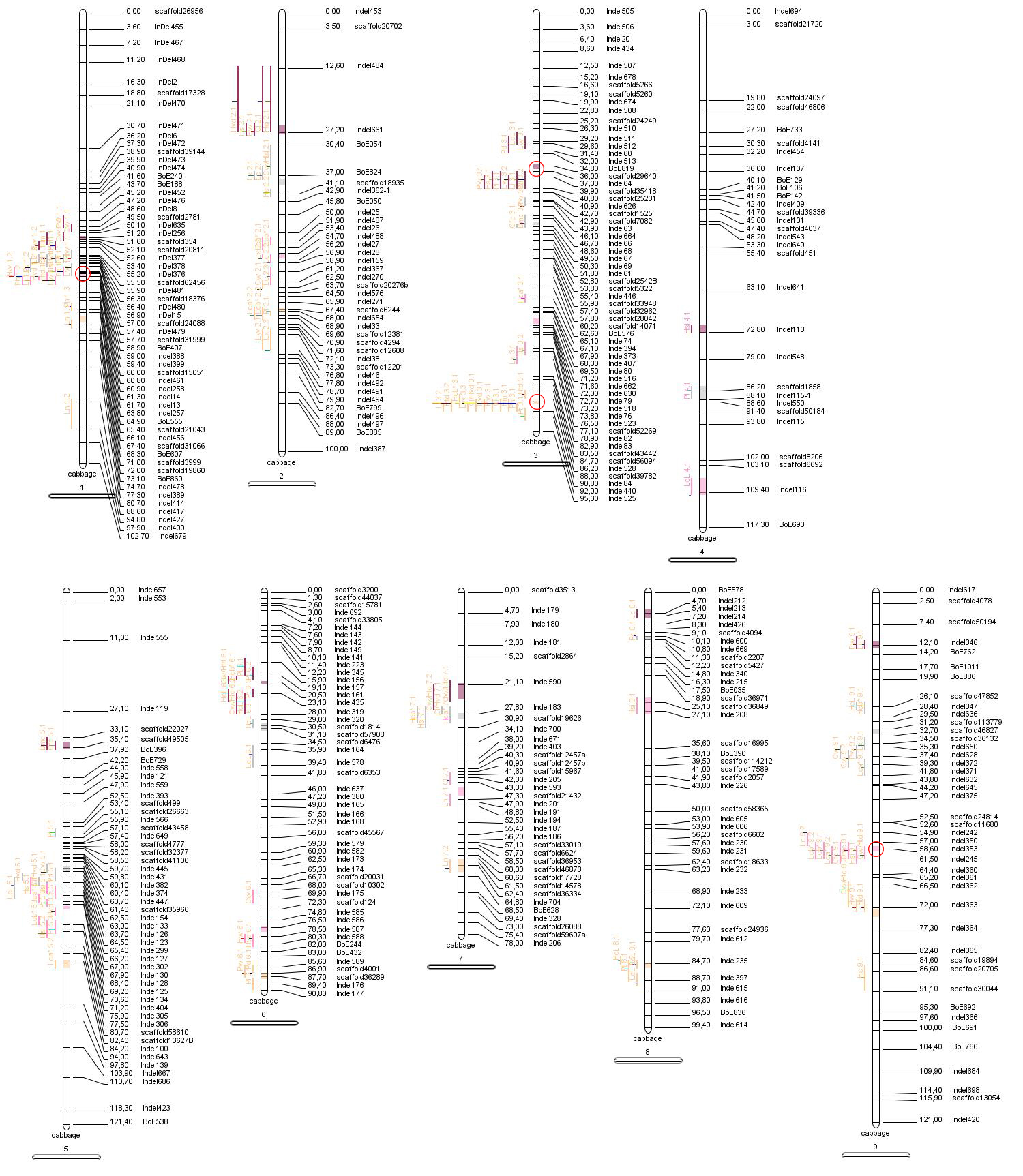

Supplement: Supplementary Figure 2 — Meta-QTL analysis for the obtained 144 QTLs. [file Image2.JPEG]
